# Probing Hydrogen Activation in a Dimetal Dihydride Complex by Symmetric Exchange with Parahydrogen

**DOI:** 10.1021/jacs.5c18194

**Published:** 2026-02-12

**Authors:** Julius F. Matz, Lukas Kaltschnee, Sara I. Mozzi, Gonzalo G. Rodriguez, Anton Römer, Ricardo A. Mata, Ilya Kuprov, Franc Meyer, Stefan Glöggler

**Affiliations:** † NMR Signal Enhancement Group, 28282 Max Planck Institute for Multidisciplinary Sciences, Am Faßberg 11., 37077 Göttingen, Germany; ‡ Center for Biostructural Imaging of Neurodegeneration, University Medical Center Göttingen, Von-Siebold-Str. 3A, 37075 Göttingen, Germany; § Institute for Inorganic Chemistry, 9375 Georg-August-Universität Göttingen, Tammannstrasse 4, 37077 Göttingen, Germany; ∥ Institute for Physical Chemistry, Georg-August-Universität Göttingen, Tammannstrasse 6, 37077 Göttingen, Germany; ⊥ Department of Chemical and Biological Physics, Weizmann Institute of Science, Rehovot 7610001, Israel; # School of Chemistry and Chemical Engineering, University of Southampton, Southampton SO17 1BJ, United Kingdom; ∇ Advanced Imaging Research Center, 34976 The University of Texas Southwestern Medical Center, Dallas, Texas 75390, United States; ○ Department of Biomedical Engineering, The University of Texas Southwestern Medical Center, Dallas, Texas 75390, United States

## Abstract

We report an investigation into the hydrogen exchange mechanism in a dinickel­(II) dihydride complex with *C*_2v_ symmetry. To obtain atomic level information on the low-concentration intermediate species, we use parahydrogen in combination with nuclear magnetic resonance (NMR). Unexpectedly, despite the chemical equivalence of the two hydride sites imposed by the mirror symmetry of the model, we observe spontaneous conversion of the nuclear singlet order into in-phase longitudinal magnetization–something that normally requires sophisticated pulse sequences. The magnetization is retained upon reductive elimination of dihydrogen from the dihydride species and observed as an enhanced hydrogen NMR signal. This is explained by a chain of nuclear relaxation interference processes that do not require asymmetric intermediates; we confirm this in two ways: using analytical relaxation theory and with brute-force numerical simulations.

## Graphical abstract

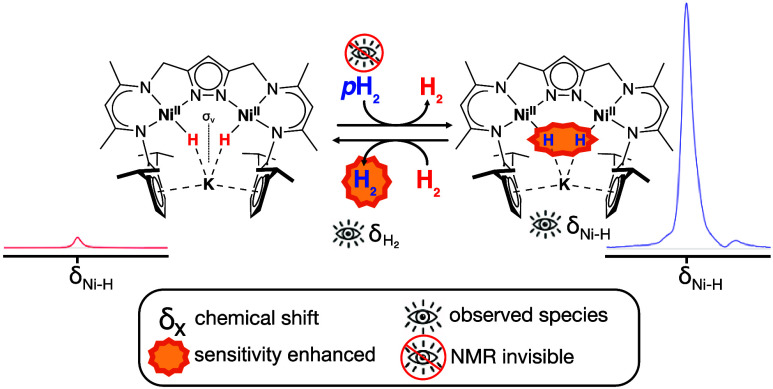

## Introduction

Understanding fundamental chemical processes observed in nature, such as hydrogen production and nitrogen fixation, is important for the development of more efficient and selective catalysts.^1,2^ This is particularly true for organometallic and enzymatic catalysis^3–6^ where transient chemical species can occur in low concentrations, but may still determine reaction pathways and the selectivity of the catalytic process. Direct observation and structural characterization of these intermediates using mainstream analytical methods is difficult and limited to special cases; there are very few generally applicable tools.^7–9^

One popular technique is nuclear magnetic resonance (NMR) spectroscopy; it is highly chemically selective and can provide structural information.^10–13^ However, NMR has low sensitivity and often struggles to detect low-concentration reaction intermediates. One way to remedy this is parahydrogen-induced polarization (PHIP)–a method with nearly 40 years of history in the study of catalytic processes.^14–16^ It belongs to the broader group of spin hyperpolarization methods^17^ and uses parahydrogen (*p*H_2_)–an easily obtainable nuclear spin isomer of molecular hydrogen.^18^ The two protons in *p*H_2_ are 100% spin-polarized relative to each other, but the total spin of two antiparallel protons is zero and the state is therefore initially NMR-silent. Chemical manipulation is therefore necessary to break the symmetry and convert that relative spin polarization into much stronger observable magnetization than what is available in conventional NMR spectroscopy.^19^

Major work in PHIP methodology today is concentrated in biomedical applications,^20^ such as cancer imaging,^21,22^ metabolomics,^23,24^ as well as in the studies of catalytic processes involving hydrogen activation.^25–27^ Even the mere presence of PHIP is informative: the mechanism must keep the two protons together to retain their spin correlation.^14,15,19^ This was initially limited to cis-hydrogenation but was later expanded to include trans-selective and geminal hydrogenation mediated by ruthenium carbene complexes.^28,29^ Those mechanistic studies later enabled hyperpolarization of fumarate^30^ and the detection of fumarase activity in cells.^31^ Since detectable magnetization is produced as soon as the *p*H_2_ symmetry is broken, the investigation of metal dihydrogen activation is possible as demonstrated by the detection of transient classical^32–34^ and nonclassical metal dihydride complexes,^35,36^ also by indirect detection through partially negative line (PANEL) experiments.^37^ Other applications include frustrated Lewis pair molecular tweezers that function as metal-free hydrogenation catalysts.^38–40^

Recent extensions of PHIP methodology have enabled mechanistic studies of hydrogenases under catalytic conditions, leading to the characterization of two previously elusive intermediates in the catalytic cycle of [Fe]-hydrogenases.^41^ These metalloenzymes^42^ are involved in H_2_ activation and production; they serve as blueprints for the development of eco-friendly catalysts.^43^ Transient diamagnetic hydrogen-bound intermediates in other metalloenzymes are now within reach, for example nitrogenases wherein metal hydrides are present in the FeMo cofactor and used to store reducing equivalents, enabling reductive binding of dinitrogen to the Fe/S active site accompanied by hydrogen release.^44–47^

In this communication, we study similar processes in dinickel dihydride complex K­[L­(Ni^II^–H)_2_] (**1H**_**2**_, where L is a compartmental ligand scaffold) that may be viewed as a model of the enzymatic reactions discussed above, because it is known to undergo replacement of the two hydride ligands with concomitant reductive binding of small molecules coupled to H_2_ release^48–53^ (see Figure 1), and because its oxidation triggers even the H_2_-releasing reductive activation of N_2_.^54^ The H_2_/D_2_ exchange reaction proceeds in a pairwise synchronous fashion without H/D scrambling,^48^ and density functional theory (DFT) calculations and characterization of the proposed {LNi^I^} intermediate suggest that this may follow a dissociative stepwise mechanism.^48,55^ In this context, parahydrogen could help confirm the proposed mechanism through NMR signal enhancement in low-concentration intermediates, and could provide evidence for the dissociative exchange pathway.

**Figure 1:**
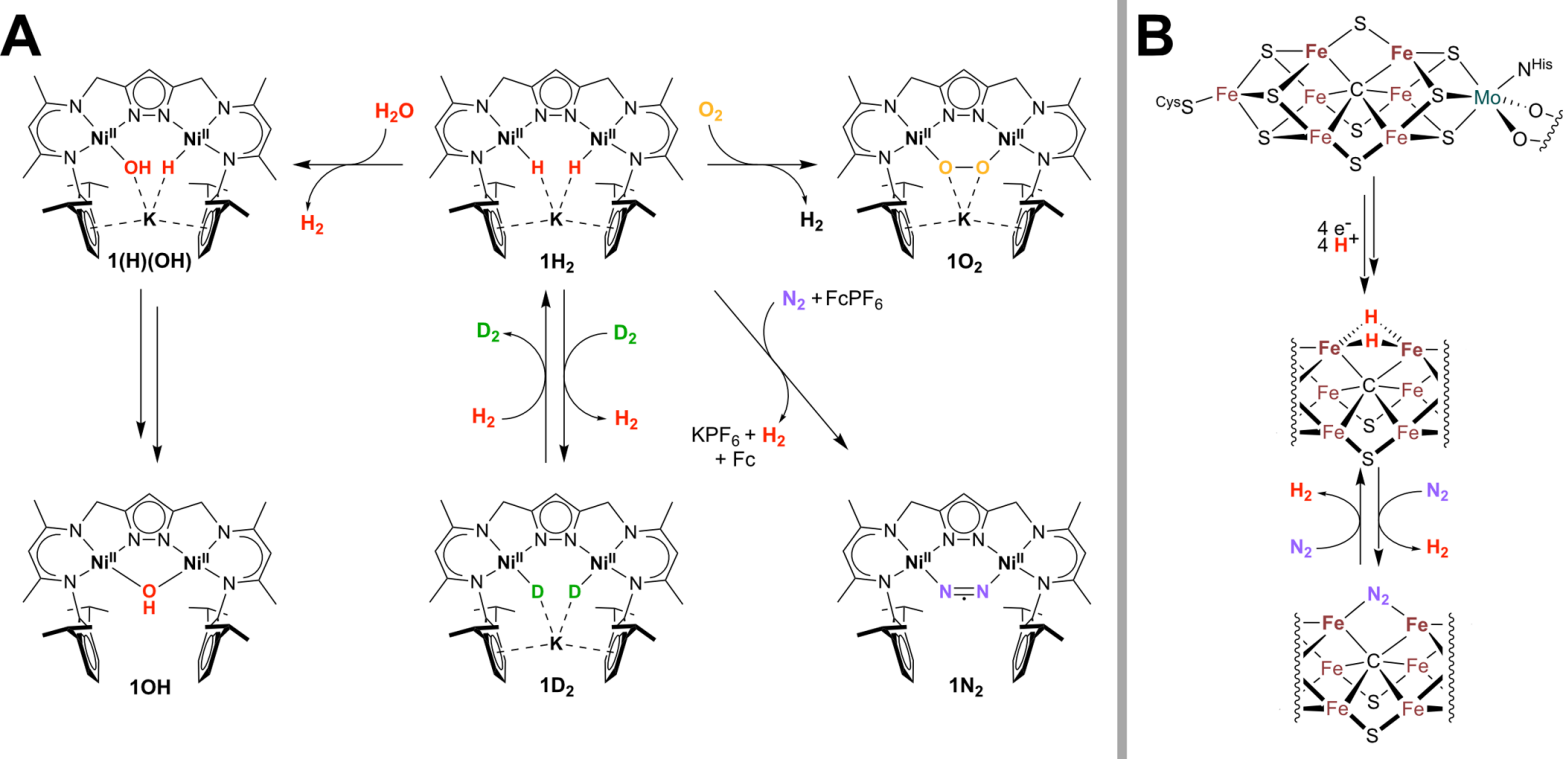
(A) Dinickel dihydride complex K­[L­(Ni^II^–H)_2_] (**1H**_**2**_) studied within this work showing pairwise H_2_/D_2_ exchange.^48^ **1H**_**2**_ is highly sensitive toward O_2_ forming the peroxido complexK­[LNi^II^_2_(O_2_)] (**1O**_**2**_) irreversibly,^49^ and toward water reacting to K­[LNi^II^_2_(H)­(OH)] (**1**(**H**)­(**OH**)) and ultimately to K­[LNi^II^_2_(μ–OH)] (**1OH**).^53^ Oxidation of **1H**_**2**_ enables reductive binding of N_2_ coupled to H_2_ release giving K­[LNi^II^_2_(μ_1,2_–N_2_)] (**1N**_**2**_);^54^ a scenario resembling the reactivity of nitrogenase metalloenzyme where binding of N_2_ to the FeMo metal cofactor is also paired with reductive elimination of H_2_ (see B).^56,57^ The hydride arrangement as well as binding mode of N_2_ in nitrogenases is still under discussion.^58–60^ Reproduced from ref 54. Copyright 2025 American Chemical Society.

The difficulty in using PHIP to elucidate the H_2_ binding mechanism of **1** is that the two hydrides in **1H**_**2**_ occupy chemically equivalent sites related by mirror symmetry. Accordingly, in the absence of structural perturbations, the two nuclear spins ought to remain in the singlet state, rendering them NMR silent under standard PHIP mechanisms.^18,35,61,62^ However, we demonstrate here that spontaneous polarization transfer from the initial parahydrogen singlet spin order to in-phase proton longitudinal magnetization does nonetheless occur. This is unexpected, but may be explained using a nuclear spin relaxation model taking into account chemical shift anisotropy (CSA) and dipole-dipole (DD) coupling interference effects–we demonstrate this both algebraically and numerically below. This CSA―CSA and DD―CSA interference mechanism bypasses the classical requirement for asymmetric intermediates.^63,64^

PHIP-CEST^37,41^ (chemical exchange saturation transfer experiments) with **1H**_**2**_ indicates the absence of asymmetric intermediates in the exchange reaction and that dihydrogen acquires net longitudinal magnetization upon release from the complex. This provides further insight into the dynamics of the H_2_ exchange process at the molecular level. Our results highlight a novel pathway for the generation of longitudinal magnetization from parahydrogen in systems where the hydrogen atoms remain chemically equivalent, emphasizing the role of nuclear spin relaxation interference effects in the dynamics of parahydrogen singlet order.

## Results

Studying air and moisture sensitive compounds requires manipulation under an inert atmosphere. Previous PASADENA-type experiments with **1H**_**2**_ were hampered by insufficiently inert conditions.^14^ Consequently, a new PASADENA setup that fulfills these specifications and that is easy to operate and maintain is crucial. Within this article, we present the development and construction of such a setup operating under anoxic and anhydrous conditions (see SI for further details).

PHIP experiments under inert conditions were carried out by preparing samples under an argon atmosphere in a glovebox. The sample holder was subsequently transferred into the bore of the magnet. The atmosphere of the operational section of the setup was repeatedly evacuated and backfilled with inert gas (nitrogen) to ensure the exclusion of air and moisture. After this purging process, the valves to the sample holder were opened, and the experiments were initiated. *p*H_2_ was introduced to the sample either automatically via computer-controlled magnetic valves or manually by operating the valves by hand.

Given the molecular symmetry of **1H**_**2**_ we were intrigued to observe enhancement of signals in the ^1^H NMR spectrum after bubbling *p*H_2_ through a sample of **1H**_**2**_ under argon (pulse sequence is depicted in SI Figure 7). In the *C*_2v_ symmetric complex **1H**_**2**_ the two hydrides occupy chemically equivalent positions related by mirror symmetry (Figure 2A). Only minor amounts of water decomposition products (88% **1H**_**2**_, 12% **1**(**H**)­(**OH**)) were detected before bubbling. This shows that the sample holder is gastight on the time scale of the experiment and is well suited for use with highly sensitive compounds. Even after bubbling with *p*H_2_ for 10 s the amounts of decomposition products increases only slightly (81% **1H**_**2**_, 12% **1­(H)­(OH)**, 7% **1O**_**2**_). After bubbling with *p*H_2_ moderate enhancements of the signals at −24.1 ppm (*I*_PHIP_/*I*_thermal_ = 9.2, 0.03% polarization) and 4.54 ppm are visible in the NMR spectrum of **1H**_**2**_ in THF-*d*_8_ although the hydrogen atoms occupy chemically equivalent positions (see Figure 2B). The former signal is assigned to the dihydride position of **1H**_**2**_ whereas the latter signal assignment has some ambiguity that will be addressed below.

**Figure 2:**
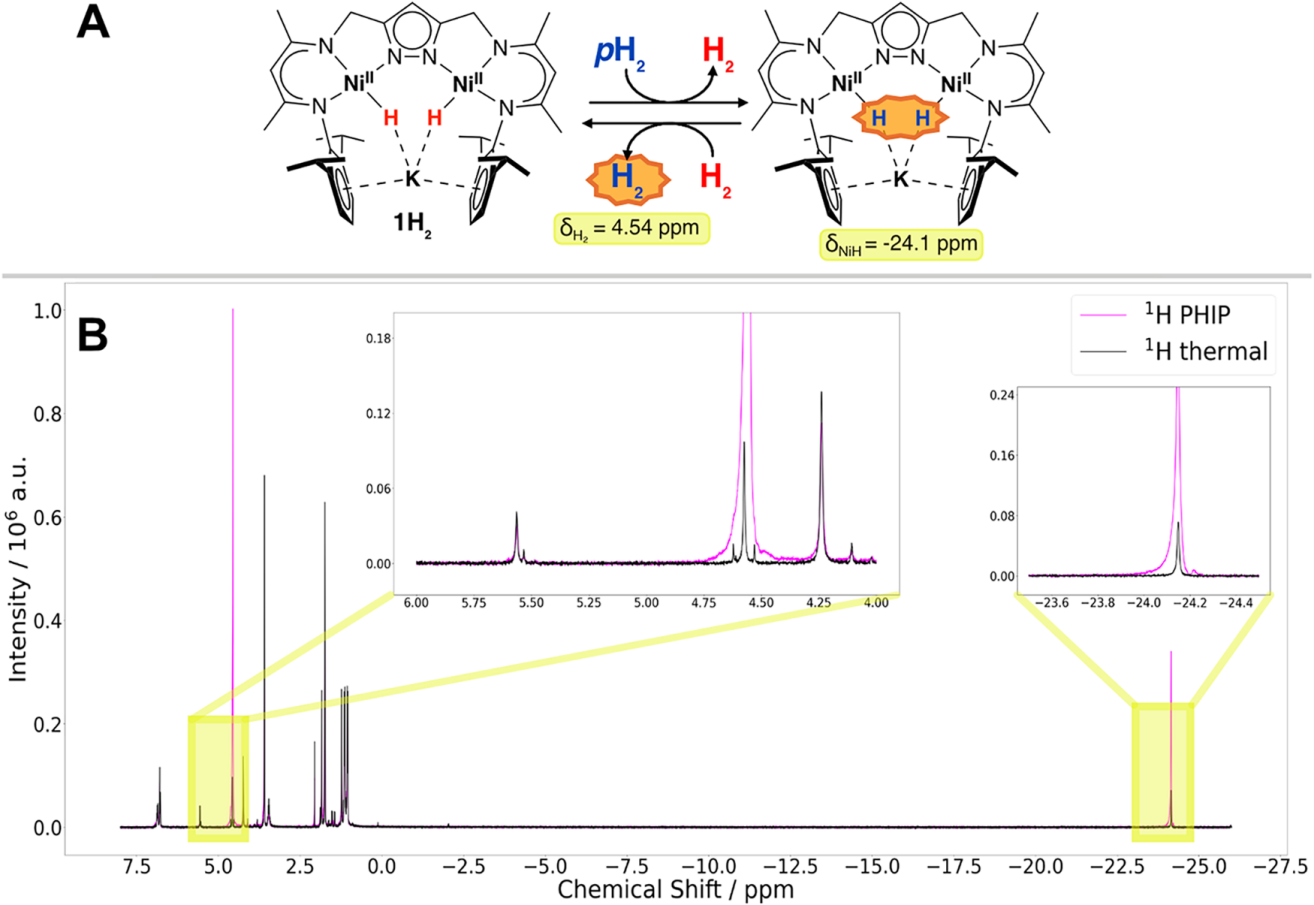
(A) Pairwise exchange of dihydride ligands of complex **1H**_**2**_ with *p*H_2_ or thermally polarized H_2_ showing enhanced signals for the dihydride and for free H_2_. (B) Overlay of ^1^H-PHIP-NMR spectra of complex **1H**_**2**_ (13.4 mM) in THF-*d*_8_ at 298 K with π/4 detection pulses before (black) and after 10 s bubbling with *p*H_2_ (magenta) showing *p*H_2_ induced enhancement of signals at 4.54 ppm and −24.1 ppm with pure in-phase magnetization. The inserts show subsections of the spectra showing sensitivity enhancements.

Notably, both signals are purelyin-phase and not antiphase as expected for PHIP experiments under PASADENA conditions.^14,37^ To emphasize the absence of classical antiphase PASADENA signals in Figure 2 we decided to use π/4 pulses for detection, even if π/2 pulses would yield the highest intensity for the in-phase PHIP signals observed. To verify that these signals originate from **1H**_**2**_, control experiments were performed under identical conditions using some of the decomposition products. Accordingly, NMR samples of **1OH** and **1O**_**2**_ were prepared independently under argon in THF-*d*_8_ and exposed to *p*H_2_ (7 bar). After 10 s of bubbling and a 2 s settling time, π/4 flip angle ^1^H NMR spectra were acquired (see Figures S9 and S10). No signal enhancement was detected in either experiment, confirming that **1H**_**2**_ is essential for the PHIP effects leading to signal enhancement.

By combining PHIP techniques with the chemical exchange saturation transfer (CEST),^65–67^ herein referred to as PHIP-CEST^41,68,69^ transiently formed species can be indirectly detected through their chemical exchange with more abundant species. This method was recently used to reveal H_2_-bound states in hydrogenase catalysis with extremely high sensitivity.^41^ Herein, we use PHIP-CEST to determine the origin of the signal at 4.54 ppm and test the hypothesis of chemical exchange between the dinickel dihydride species (−24.1 ppm) and free hydrogen. The hydride region in the NMR is tested (−25 ppm as a high-field limit) to exclude the presence of possible intermediates on the exchange reaction pathway. When applying a radio frequency (RF) spin-lock pulse on resonance with a signal of a certain species, the corresponding NMR transition is saturated, and the signal is attenuated. In CEST, saturation is transferred from one species to another if both are related by chemical exchange. In the system under study, the signal at 4.54 ppm can be attributed either to the methylene proton of the β-ketiminato ligand backbone of **1H**_**2**_ or to free H_2_, presumably polarized by exchange with *p*H_2_ bound transiently to the nickel complex. To provide evidence that the signal at 4.54 ppm stems from freely dissolved H_2_, 24 ^1^H NMR spectra with π/4 flip-angle were acquired following the pulse sequence depicted in Figure S11. The saturation irradiation was applied for 2 s with a spin-lock amplitude of 50 Hz and central frequencies varied according to lists given in the SI. Accordingly, a reduction of the dihydrogen signal at 4.54 ppm is expected when irradiating at −24.1 ppm. By plotting the integral of these signals against the continuous wave (CW) irradiation offset, PHIP-CEST profiles are obtained (see Figure 3).

**Figure 3:**
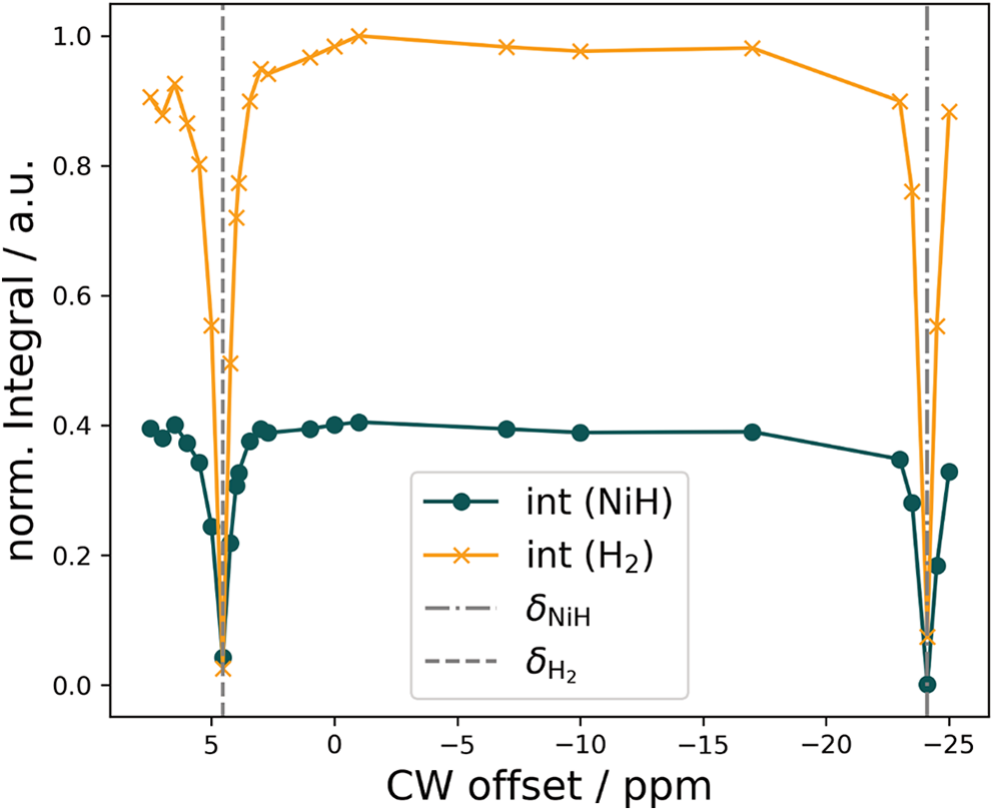
^1^H-PHIP-CEST profiles obtained for **1H**_**2**_ (13.4 mM) in THF-*d*_8_. The value of the Ni–H integral at −24.1 ppm (green) or H_2_ integral at 4.54 ppm (orange) in a.u. is plotted vs the CW irradiation offset in ppm relative to tetramethyl silane. PHIP-CEST profiles were reproduced more than 3 times with different samples and spin-lock-frequency lists (see Figures S12–S15). NMR-invisible *p*H_2_ exchanges with the dihydride complex, leading to sensitivity enhancement of the dihydride signal at −24.1 ppm. CW irradiation at this offset leads to a sharp decrease in signal intensity at 4.54 ppm (and vice versa) indicative of polarized free H_2_ in solution. All PHIP-CEST spectra were acquired with π/4 flip-angle pulses as single-scan spectra at 9.4 T (400 MHz) using 2 s cw saturation after 10 s bubbling and allowing the sample to settle for 1 s according to Figure S11. Spin-lock-field amplitudes (|γ_H_ *B*_1_|) were set to 50 Hz and spin-lock-field offsets (cw offset) were varied according to uniformly and non-uniformly distributed lists of 24 points from −25 to 7.5 ppm.

## Discussion

When irradiating at the hydride position, the signal intensity at 4.54 ppm decreases dramatically and vice versa. This indicates that the signal at 4.54 ppm belongs to free hydrogen, which was released from **1H**_**2**_ by reversible reductive elimination, in accordance with the mechanism for the pairwise H_2_/D_2_ exchange.^48^ Herein, *p*H_2_ presumably exchanges with the hydride ligands in **1H**_**2**_, is converted to *o*H_2_ (orthohydrogen) upon singlet–triplet transition, and is released in a polarized state by reductive elimination, leaving behind an unsaturated complex able to bind hydrogen again.

Sophisticated pulse sequences^70^ and chemical transformations^19,71^ are normally required to convert nuclear singlet order into magnetization, but the process can also happen spontaneously through the dipole-dipole/chemical shift anisotropy (DD-CSA) interference mechanism^72^ which opens cross-relaxation channels from the longitudinal correlation component **I**_Z_ **S**_Z_ of the singlet state to longitudinal magnetization states **I**_Z_ and **S**_Z_. This mechanism is significant when the **I**_±_ **S**_∓_ part of the singlet disappears sufficiently fast.^63,64^

In this instance, we have a two-proton system with a *J*-coupling, a dipole-dipole coupling, and two noncollinear chemical shift tensors not aligned in the spin-spin direction

1 $$H=I\cdot Z_{I}\cdot B+S\cdot Z_{S}\cdot B+2\pi JI\cdot S+I\cdot D\cdot S$$

where **B** is the magnetic field vector (Tesla), **I** and **S** are vectors of three Cartesian projection operators for the two spins (dimensionless), **Z** = −γ­(**1**–**σ**) are Zeeman interaction tensors (rad/s·T), including the isotropic Zeeman interaction with a magnetogyric ratio γ and the chemical shielding **σ**; *J* is the scalar coupling constant (Hz), and **D** is the dipole-dipole coupling tensor (rad/s). In a strong vertical magnetic field **B** = [0 0 *B*_0_]^T^, the isotropic part of this Hamiltonian

2 $$\begin{array}{l} H_{0}=\omega_{I}I_{Z}+\omega_{S}S_{Z}+2\pi JI\cdot S\\ \omega_{I,S}=B_{0}\mathrm{Tr}\left(Z_{I,S}\right)/3 \end{array}$$

is symmetric with respect to the permutation of the two spins because here ω_I_ = ω_S_. The singlet state density matrix commutes with this Hamiltonian and therefore cannot evolve. However, the two Zeeman tensors **Z**_I,S_ are only related by mirror symmetry–not by inversion or permutation symmetry, meaning that relaxation leakage is expected^73^ from the singlet subspace.

Automatic symbolic evaluation^74^ of Bloch-Redfield-Wangsness relaxation superoperator^75,76^

$$\begin{array}{l} R=-\left\langle \int_{0}^{\infty }H_{1}\left(0\right)e^{-iH_{0}\tau }H_{1}\left(\tau \right)e^{+iH_{0}\tau }d\tau \right\rangle \\ H\rho =\left[H,\rho \right] \end{array}$$ 3

in the case of isotropic rotational diffusion (see the *Mathematica* script in the Supporting Information) reveals that the cross-relaxation rate from the pure singlet state to **I**_Z_ + **S**_Z_ is zero, but that longitudinal and transverse components of the singlet have different relaxation rates 
4

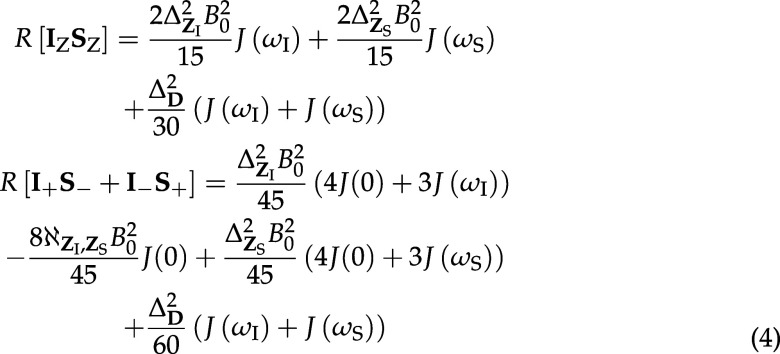

Here, the invariants of 3 × 3 Cartesian interaction tensors are defined as^77^

5

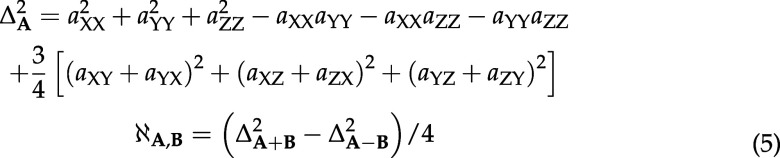

and *J*(ω) = τ_C_/(1 + ω^2^τ_C_^2^) is the isotropic rotational diffusion autocorrelation function.

The presence of *J*(0) terms in *R*[**I**_+_ **S**_–_ + **I**_–_ **S**_+_] means that the transverse components of the singlet disappear faster than the longitudinal components. This breaks the symmetry lockout and allows longitudinal magnetization to accumulate via DD-CSA cross-correlation with the following rate 
6

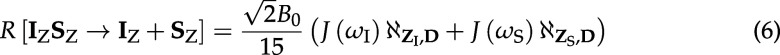

This process is easily visualized (Figure 4) in *Spinach* (see the *Matlab* script in the Supporting Information), where the Bloch-Redfield-Wangsness theory module is designed to account for all known and unknown Redfield type processes of all ranks automatically.^77,78^

**Figure 4:**
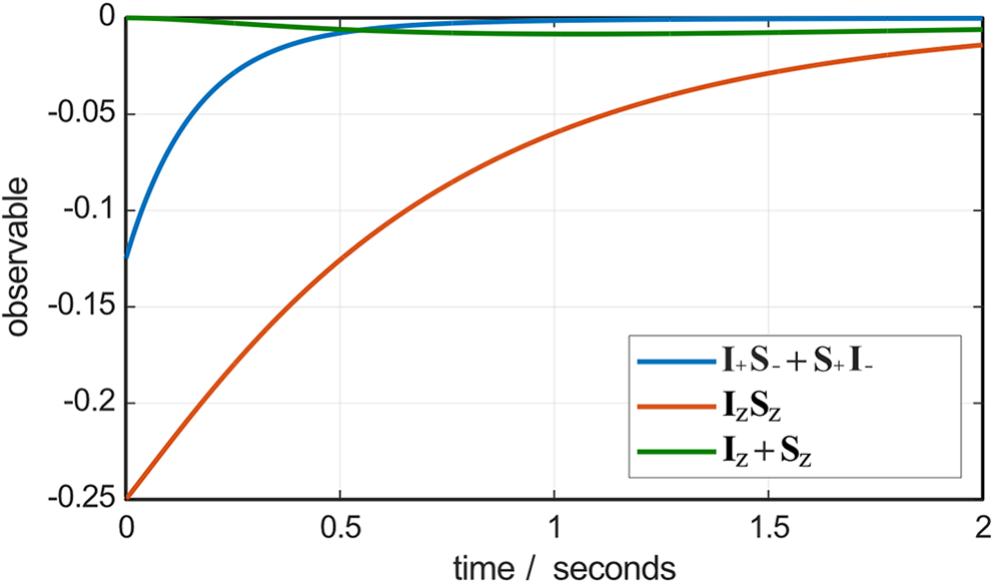
Numerical simulation, using *Spinach* 2.9,^79^ of the dynamics of the components of the singlet state (blue and red traces) and longitudinal magnetization (green trace) for a system initially in a pure singlet state and evolving in time under the action of the Hamiltonian and the Bloch-Redfield-Wangsness relaxation superoperator assuming a rotational correlation time of 0.5 ns and magnetic field of 18.79 T. The dipole-dipole coupling tensor was obtained from the energy minimum molecular geometry (CPCM THF) obtained using PBE0-D3­(BJ)/def2-TZVP method in ORCA.^80^ Proton chemical shielding tensors were then obtained using GIAO method and def2-TZVPP basis set in the same program.

## Conclusion

We have observed parahydrogen-induced NMR signal enhancements for a *C*_2v_ symmetric dinickel dihydride complex that couples the reductive activation of challenging substrates to H_2_ release, akin to the proposed mechanism for nitrogenase enzymes. We report an experimental setup that enables PASADENA-type PHIP experiments for sensitive compounds under inert conditions.

The unusual spontaneous interconversion of the nuclear singlet order into in-phase longitudinal magnetization, apparently without symmetry breaking, was explained by a chain of nuclear spin relaxation interference effects: first CSA-CSA cross-correlation makes longitudinal and flip-flop components of the singlet relax at different rates, and then DD-CSA cross-correlation converts longitudinal two-spin order into longitudinal magnetization. The presence of longitudinally magnetized dihydrogen was corroborated by PHIP-CEST experiments; no asymmetric intermediates were found.

Although in general, contributions of paramagnetic intermediates cannot be excluded, there is no direct evidence for such contributions, since the DD-CSA cross-correlated relaxation mechanism explains our observations. In analogy to recent PHIP experiments employing phosphorus biradicaloids,^81^ the putative {LNi^I^} intermediate likely also does not interfere with PHIP generation due to its singlet electronic ground state.^55^

Our findings suggest that parahydrogen techniques can be applied to symmetric compounds, improving NMR sensitivity and facilitating the study of symmetric molecules in catalysis and materials science. Further work on this methodology will enable the investigation of hydrogen-bound intermediates in hydrogenase and possibly nitrogenase enzymes, as well as their synthetic model systems.

## Supplementary Material











## Notes

Experimental Methods, ^1^H NMR spectra of relevant compounds, additional PHIP-CEST profiles and technical drawings (PDF)
Mathematica script for a CSA-DD-CSA system, complete relaxation analysis (NB)
List of the parts used for construction of the inert Bubbling Setup (XLSX)
PHIP-CEST pulse programm including exemplary data (ZIP)
Matlab script: time-dependence of **I**_*z*_ **S**_*z*_ and **I**_*z*_ + **S**_*z*_ spin orders in a parahydrogen molecule coordinated to a nickel cage (m) (TXT)
