# Supplementary material for: Probing Hydrogen Activation in a Dimetal Dihydride Complex by Symmetric Exchange with Parahydrogen
Source: J Am Chem Soc. 2026 Feb 12;148(7):7181–8. doi: 10.1021/jacs.5c18194 (PMC12951439; doi:10.1021/jacs.5c18194)
Supplement: Supplementary file 1 [file ja5c18194_si_001.pdf]

Supporting Information: Probing Hydrogen Activation in a Dimetal Dihydride Complex by Symmetric Exchange with Parahydrogen

Julius F. Matz,[†,‡] Lukas Kaltschnee,[†,‡] Sara I. Mozzi,[¶] Gonzalo G. Rodriguez,[†,‡] Anton Römer,[§] Ricardo A. Mata,[§] Ilya Kuprov,[‖,⊥] Franc Meyer,[¶] and Stefan Glöggler*,[†,‡,#,@]

†*NMR Signal Enhancement Group, Max Planck Institute for Multidisciplinary Sciences, Am Faßberg 11, 37077 Göttingen, Germany*

‡*Center for Biostructural Imaging of Neurodegeneration, University Medical Center Göttingen, Von-Siebold-Str. 3A, 37075 Göttingen, Germany*

¶*Institute for Inorganic Chemistry, Georg-August-Universität Göttingen, Tammannstrasse 4, 37077 Göttingen, Germany*

§*Institute for Physical Chemistry, Georg-August-Universität Göttingen, Tammannstrasse 6, 37077 Göttingen, Germany*

‖*Department of Chemical and Biological Physics, Weizmann Institute of Science, Rehovot 7610001, Israel*

⊥*School of Chemistry and Chemical Engineering, University of Southampton, Southampton SO17 1BJ, United Kingdom*

#*Advanced Imaging Research Center, The University of Texas Southwestern Medical Center, Dallas, Texas 75390, United States*

@*Department of Biomedical Engineering, The University of Texas Southwestern Medical Center, Dallas, Texas 75390, United States*

E-mail: stefan.gloeggler@mpinat.mpg.de

Contents

1 Supplementary Methods **S-2**

1.1 NMR Spectroscopy S-2

1.2 Inert Bubbling Setup S-3

1.3 Complex Synthesis S-7

1.3.1 $K[L(Ni-H)_2]$ (**$1H_2$**) S-7

1.3.2 $K[LNi_2(\mu\text{-}OH)]$ (**1OH**) S-8

1.3.3 $K[LNi_2(O_2)]$ (**$1O_2$**) S-8

1.4 PHIP Experiments S-10

1.5 PHIP-CEST Experiments S-13

1.5.1 Additional Discussion for PHIP-CEST Experiments S-14

1.6 Additional PHIP-CEST Profiles S-15

References **S-18**

1 Supplementary Methods

1.1 NMR Spectroscopy

Thermal ^{1}H, PHIP ^{1}H and PHIP-CEST NMR spectra were recorded on a *Bruker Avance III HD 400 MHz* spectrometer at 9.4 T and 298 K unless otherwise noted. Wilmad 5 mm high throughput NMR tubes were used with the PEEK sample holder described below. Chemical shifts are reported in ppm relative to tetramethylsilane. Residual proton signals of the deuterated solvents were used as an internal reference for ^{1}H NMR spectra. THF-d_8 (3.58 ppm) used for NMR experiments was purchased from Eurisotop GmbH and dried over NaK-alloy for several days, freshly distilled before use by trap-to trap transfer *in vacuo* and stored over activated 3 Å molecular sieves inside an argon glove box. The acquired spectra were analyzed using Topspin 4.0.8 or MestreNova.

1.2 Inert Bubbling Setup

The setup consists of three main compartments, namely 1) a pH_2 generator, reservoir and general gas supply, 2) an operational part controlling the pressure, gas flow and gas switching, and 3) a detachable sample holder placed inside a NMR spectrometer. The components are interconnected by poly amide (PA) tubing, as illustrated in Figure S1.

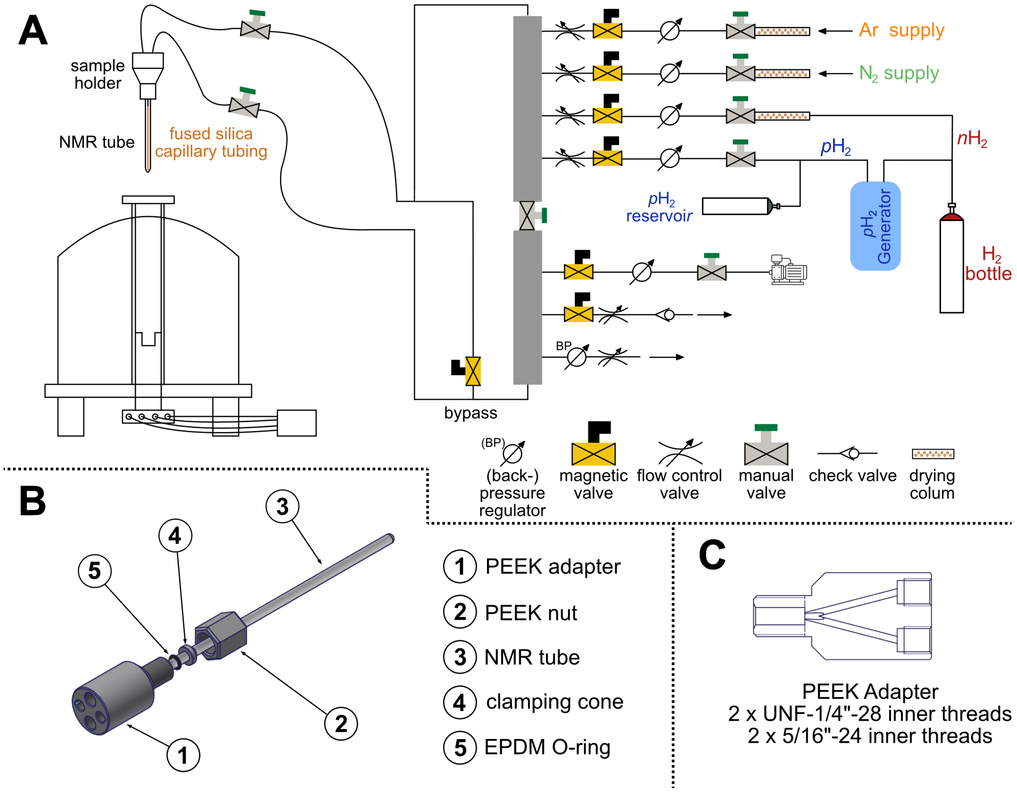

Figure S1: A) Schematic drawing of inert PASADENA Setup. Magnetic valves can be operated by TTL output signals of the NMR controller. The various gases used where dried by columns filled with molecular sieves (3 Å) and/or phosphorous pentoxide. The sample holder is connected to the operational part by 1/16" PA tubing. B) Technical drawing of the PEEK sample holder used with 5 mm NMR tubes. Inner threads allow the connection of tubing via UNF 1/4"-28 fittings. Gases are supplied to the sample by a thin fused-silica capillary (250 μm inner diameter) and pressures up to 7 bar are tolerated. C) Close-up of the sample holder PEEK adapter with up to 4 inner threads.

The initial component of the apparatus is the pH_2 generator and reservoir. The required amount of hydrogen gas (Air Liquide in 99.999% purity) is supplied to a copper coil, containing the H_2 spin conversion catalyst (Fe_2O_3), that is submerged in a liquid nitrogen

dewar (77 K) establishing the temperature-dependent ortho- to parahydrogen conversion. This enables the generation of 50% para-enriched hydrogen gas (referred to as $p\text{H}_2$ in the following) at a pressure of 12 bar in a 2 L aluminium reservoir within 20 minutes.

The various gases ($p\text{H}_2$, $n\text{H}_2$, N_2, Ar) are supplied to the operational part of the apparatus via PA tubing. Thermally polarized hydrogen gas ($n\text{H}_2$), and nitrogen gas made by evaporation of liquid nitrogen were dried by passing through ID 8 mm tubing packed with P_2O_5 or molecular sieves (3 Å) before feeding into the bubbling setup. The operational part is responsible for pressure and flow regulation of gases, their distribution and gas-switching, and resembles the central component for PHIP experiments. It consists of an array of manual shut-off valves, regulators, magnetic valves and flow-control valves mounted on an aluminium plate. The aluminium plate is supported by a height-adjustable base, allowing it to be extended from 1 m to 2.7 m, enabling it to be used with a wide range of spectrometers.

The third part of the full setup is a detachable sample holder manufactured from polyether ether ketone (PEEK). This component functions with 5 mm high-throughput NMR tubes, operating at pressures of up to 7 bar. The tubes are affixed by a tightly-fitted PEEK clamping cone and PEEK nut screwing onto the thread on the holder body. The sealing of the system is ensured by an O-ring made of ethylene propylene diene monomer (EPDM) rubber. The configuration of the holder facilitates the implementation of a variable number of drilled threads into the PEEK adapter, thereby ensuring modularity for the connection of tubing via UNF-1/4"-28 or UNF-5/16"-24 fittings and potential future expansions. Thin capillaries made from fused silica reach into the NMR tube and facilitate the bubbling. They are glued to 1.6 mm fluorinated ethylene propylene (FEP) tubing using epoxy resin. The sample holder is connected to the operational part by 1.6 mm PTFE tubing, UNF-1/4"-28 fittings and manual shut-off valves (Upchirch Scientific) with a poly(ethene-co-tetrafluoroethene) (ETFE) body and poly(chlorotrifluoroethylene) (PCTFE) rotor. This allows independent handling under inert atmosphere and gas-tight conditions, thus

enabling the preparation of analyte samples under highly controlled conditions, e.g. in glove boxes, and transport to the NMR spectrometer, making the study of highly-sensitive compounds possible. Gas tightness of the setup was checked in regular intervals during construction and before measurements by using a RS PRO PS GD-38 combustible gas leak detector, following the tubing and checking each joint while flushing the setup with $n\mathrm{H}_2$ or $p\mathrm{H}_2$. The parts used for the construction of the inert bubbling setup are listed in the attached inert_list_of_parts.xlsx file. The sample holder and y-piece for the connection of inlet line and bypass were manufactured from the mechanical workshop of the Max Planck Institute for Multidisciplinary Sciences and the technical drawings are depicted below.

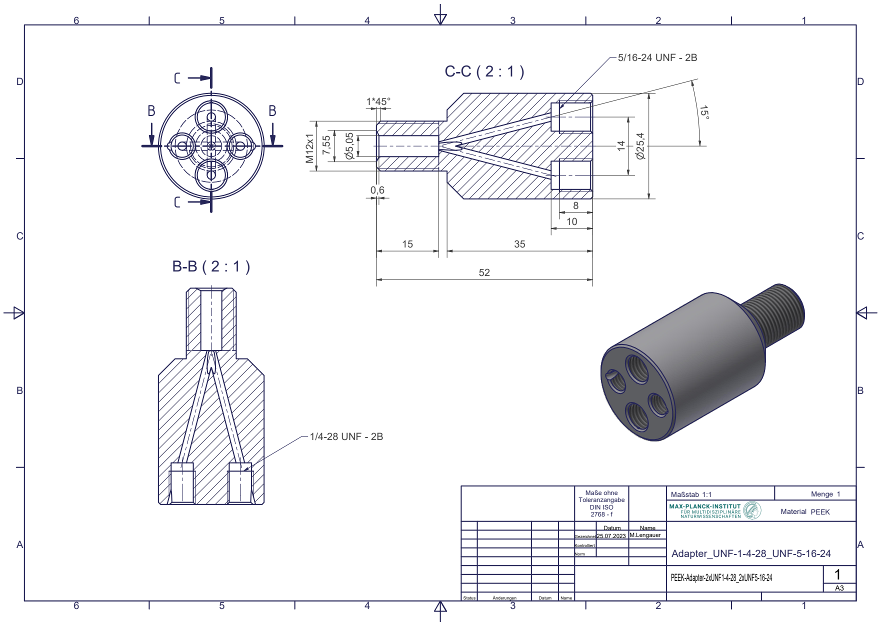

Figure S2: Technical drawing of the PEEK sample holder body with four inner threads provided and manufactured by the Mechanical Workshop of the MPI for Multidisciplinary Sciences.

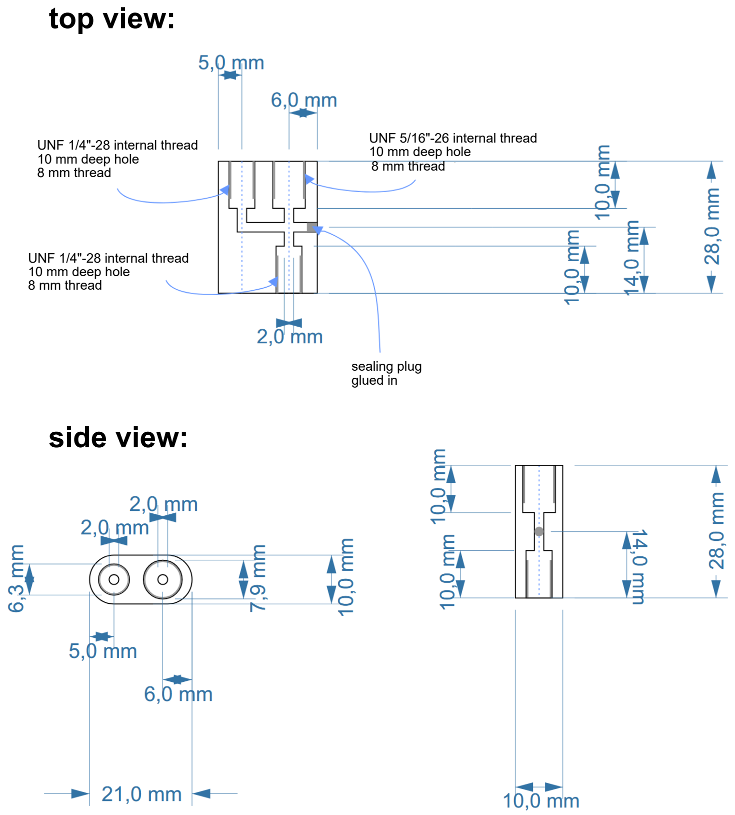

Figure S3: Technical drawing of the PEEK Y-piece for connection of bypass and inlet line. Provided and manufactured by the Mechanical Workshop of the MPI for Multidisciplinary Sciences.

1.3 Complex Synthesis

1.3.1 K[L(Ni–H)$_2$] (1H$_2$)

Complex **1H$_2$** was synthesized according to a procedure reported in literature.[S1]

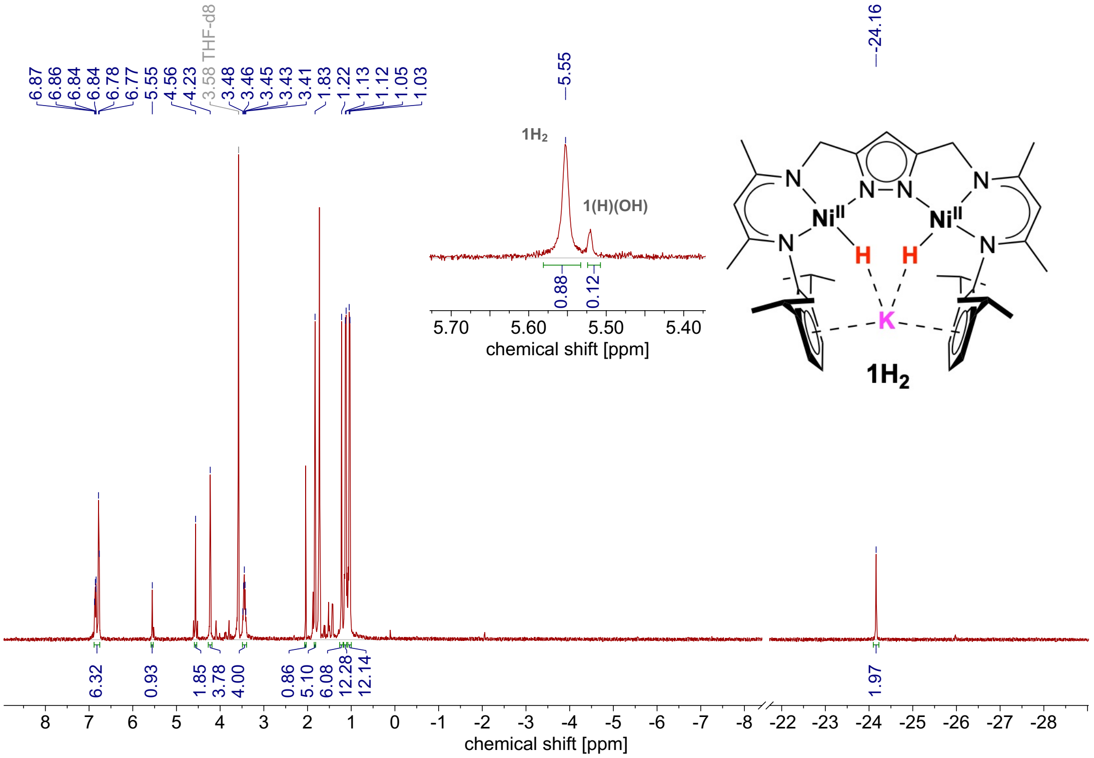

Figure S4: 90° ^{1}H NMR spectrum of **1 H$_2$** (400 MHz, THF-d_8) before bubbling experiments. The inset shows the pyrazolate backbone proton used for identification of the sample composition. In this sample: 88% **1H$_2$** and 12% **1(H)(OH)**. The sample composition before bubbling experiments varied from 88% to 94% **1H$_2$** with the other impurities **1O$_2$**, **1(H)(OH)** and **01 OH** in changing ratios depending on the amount of residual oxygen and water.

^{1}H NMR (400 MHz, THF-d_8): δ (ppm) = 7.01 – 6.61 (m, 6H, Ar–H), 5.55 (s, 1H, Pz–H), 4.56 (s, 2H, CCHC), 4.23 (s, 4H, H$_2$C–Pz), 3.45 (m, 4H, $\underline{H}$C(CH$_3$)$_2$), 1.83 (s, 6H, (HC)C(C$\underline{H}_3$)), 1.22 (s, 6H, ($\underline{H}_3$C)$_2$CH), 1.13 (d, J = 6.9 Hz, 12H), 1.04 (d, J = 6.9 Hz, ($\underline{H}_3$C)$_2$CH ,12H), −24.16 (s, 2H, Ni–H).

1.3.2 K[LNi$_2$(μ-OH)] (1OH)

Complex **1OH** was prepared according to a procedure reported in literature.[S2]

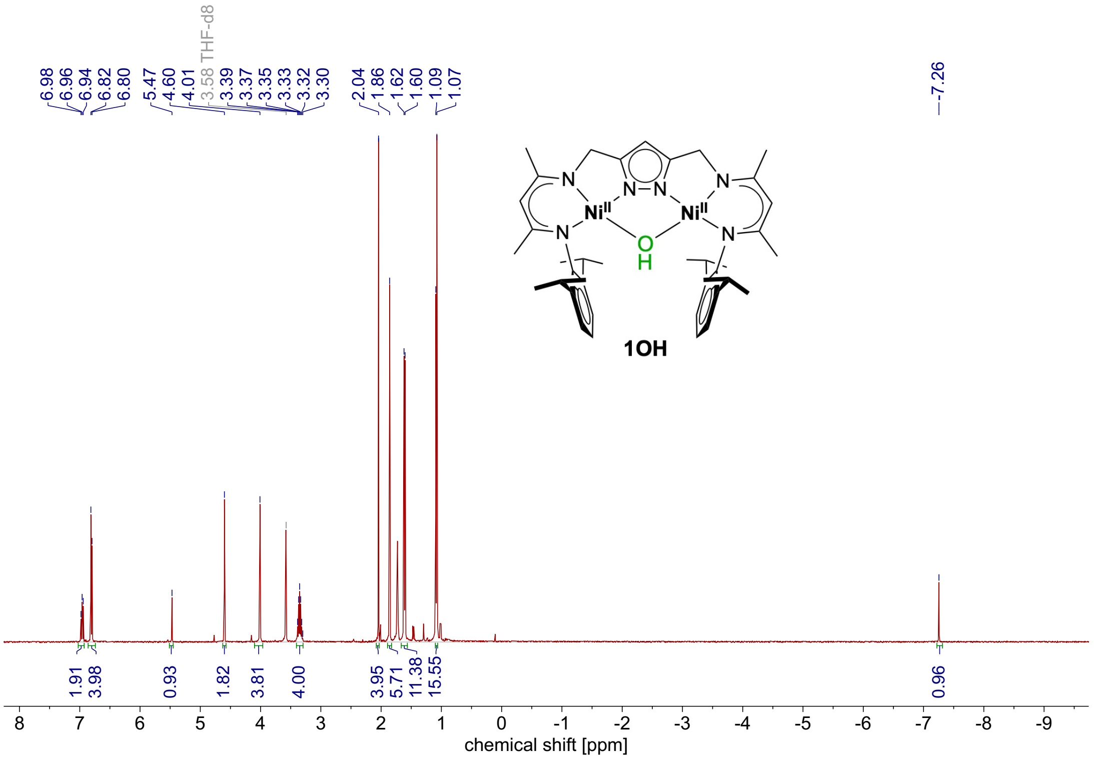

Figure S5: 90° ^{1}H NMR spectrum of **1OH** (400 MHz, THF-d_8) before bubbling experiments.

^{1}H NMR (400 MHz, THF-d_8): δ (ppm) = 6.96 (t, J = 7.6 Hz, 2H, Ar–H), 6.81 (d, J = 7.6 Hz, 4H, Ar–H), 5.47 (s, 1H, Pz–H), 4.60 (s, 2H, CCHC), 4.01 (s, 4H, H$_2$C–Pz), 3.34 (h, J = 7.0 Hz, 4H, $\underline{H}$C(CH$_3$)$_2$), 2.04 (s, 4H, (HC)C(C$\underline{H}_3$), 1.86 (s, 6H, (HC)C(C$\underline{H}_3$)), 1.61 (d, J = 7.0 Hz, 12H, ($\underline{H}_3$C)$_2$CH), 1.08 (d+s, J = 7.0 Hz, 14H, ($\underline{H}_3$C)$_2$CH + (HC)C(C$\underline{H}_3$)), −7.26 (s, 1H, Ni–OH).

1.3.3 K[LNi$_2$(O$_2$)] (1O$_2$)

The peroxo complex **1O$_2$** was obtained by preparing a low concentrated sample of K[L(Ni–H)$_2$] (**1H$_2$**) (2 mg in 500 μL THF-d_8). The residual oxygen in the tubing was

sufficient to substitute H_2 from complex **$1H_2$** and form complex **$1O_2$** as indicated by a colour change of the solution inside the argon glovebox. ^{1}H NMR analysis before bubbling with pH$_2$ through the solution indicated the presence of **$1O_2$** almost exclusively, although minor amounts of **1(H)(OH)** and **1OH** also formed and marked with an asterisk in the corresponding NMR spectrum (Figure S6).

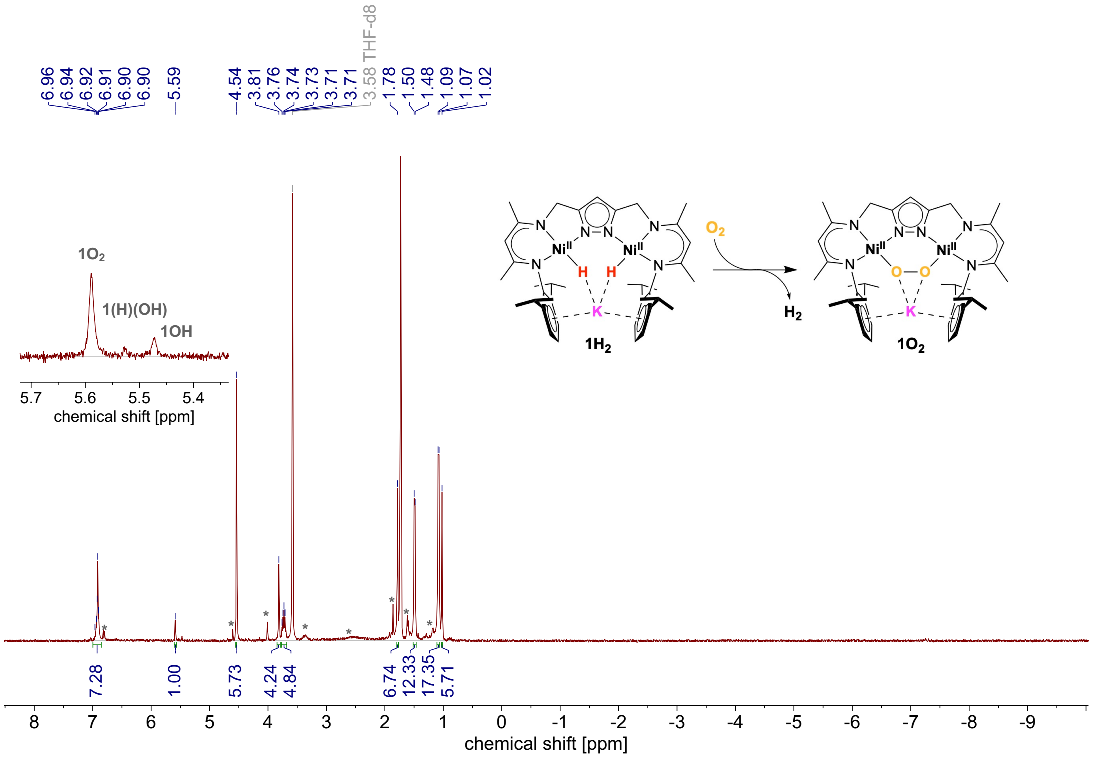

Figure S6: 90° ^{1}H NMR spectrum of **$1O_2$** (400 MHz, THF-d_8) before bubbling experiments. Identified compounds were denoted in grey as: **$1O_2$**, **1(H)(OH)**, **1OH**. The peaks labeled with an asterisk either belong to **1(H)(OH)** or **1OH** or other impurities.

^{1}H NMR (400 MHz, THF-d_8): δ (ppm) = 6.99 – 6.86 (m, 6H), 5.59 (s, 1H), 4.54 (s, 4H), 3.81 (s, 6H), 3.78 – 3.67 (m, 4H), 1.78 (s, 6H), 1.49 (d, J = 6.8 Hz, 12H), 1.08 (d, J = 6.8 Hz, 12H), 1.02 (s, 6H).

1.4 PHIP Experiments

Samples were prepared inside an argon glovebox ($O_2 < 0.5$ ppm, $H_2O < 0.5$ ppm) from Glovebox Systemtechnik in deuterated solvents using sample holders (PEEK) that were tested for gas tightness and bubbling function before each measurement. For each sample preparation the sample holders were transferred into the glovebox and disassembled with opened valves. The sample holders were assembled inside the glovebox and their tubing was flushed several times by drawing up the glovebox atmosphere with a syringe and pressing the gas through the sample holders tubing. In a 4 mL vial, the corresponding compound (**1H$_2$**, **1OH**) was dissolved in degassed THF-d_8 (500 μL in the desired concentration (13.4 mM, 17.1 mM and 18.1 mM for **1H$_2$** and 12.4 mM for **1OH**) and transferred to a heated 5 mm NMR tube. This tube was connected to the sample holder subsequently by the PEEK clamping cone and nut and sealed with a rubber o-ring. After closing the valves, the sample holders were transferred out of the glovebox and to the bubbling setup. The setup was kept pressurized with nitrogen at all times and the sample holders were connected under N_2 counterflow. By several evacuation cycles, the atmosphere within the setup was exchanged for dry nitrogen and for pH$_2$ thereafter. The NMR samples were placed inside the magnetic field, locked to the corresponding signals of the deuterated solvent and shimmed. After adjusting all acquisition parameters, thermal ^{1}H NMR spectra with a flip angle θ of 90° and 45° were collected prior to the PHIP experiments. Then, the valves connecting the sample holders to the setup were opened and the samples were pressurized to 7 bar with pH$_2$ unless otherwise noted by opening the respective magnetic valve and bypass valve manually. Bubbling was conducted for 10 s at 7 bar. $\theta = 45°$ spectra with acquisition parameters identical to the pre-spectra were measured after a settling time of 2 s according to the pulse sequence depicted in figure S7. The overlays of single-scan spectra for the different compounds with 45° flip angle acquisition were plotted using Python 3.0 in jupyter notebooks (Figures S8, S9, S10).

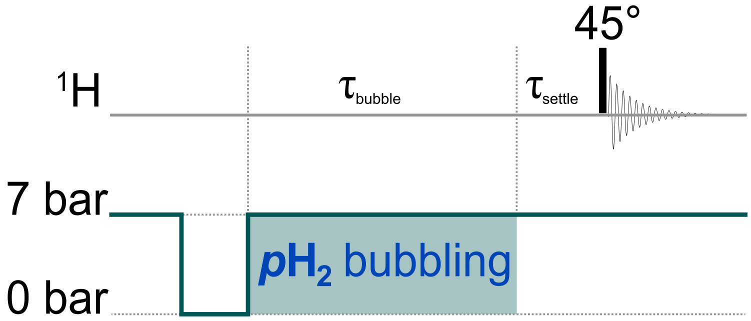

Figure S7: Pulse program used for ^{1}H-PHIP-NMR experiments with pH$_2$ bubbling for 10 s at 7 bar, 2 s settling time and 45° acquisition at 9.4 T (400 MHz).

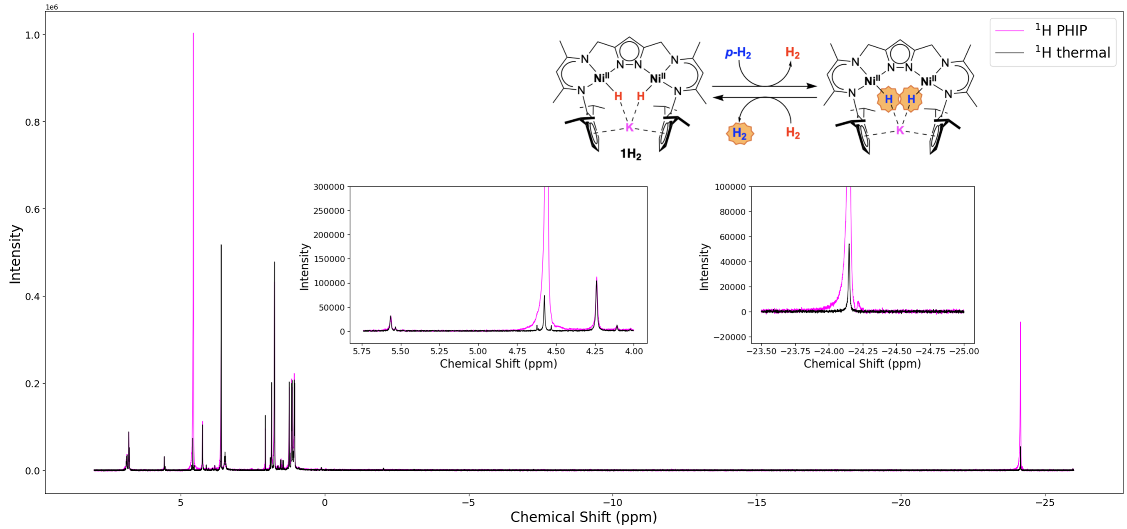

Figure S8: Overlay of 45° flip angle ^{1}H-PHIP-NMR single scan spectra of complex **1H$_2$** (5.1 mg, 13.4 mM) in THF-d_8 (500 μL) at 298 K before (black) and after 10 s bubbling with p-H$_2$ (magenta) showing pH$_2$ induced enhancement of signals at 4.54 ppm and −24.1 ppm with pure in-phase magnetisation. The inserts show zoom-in fractions of the spectral regions with sensitivity enhancements. Sample composition before the measurement: (**1O$_2$/1H$_2$/1(H)(OH)/1OH**)=0/88/12/0%.

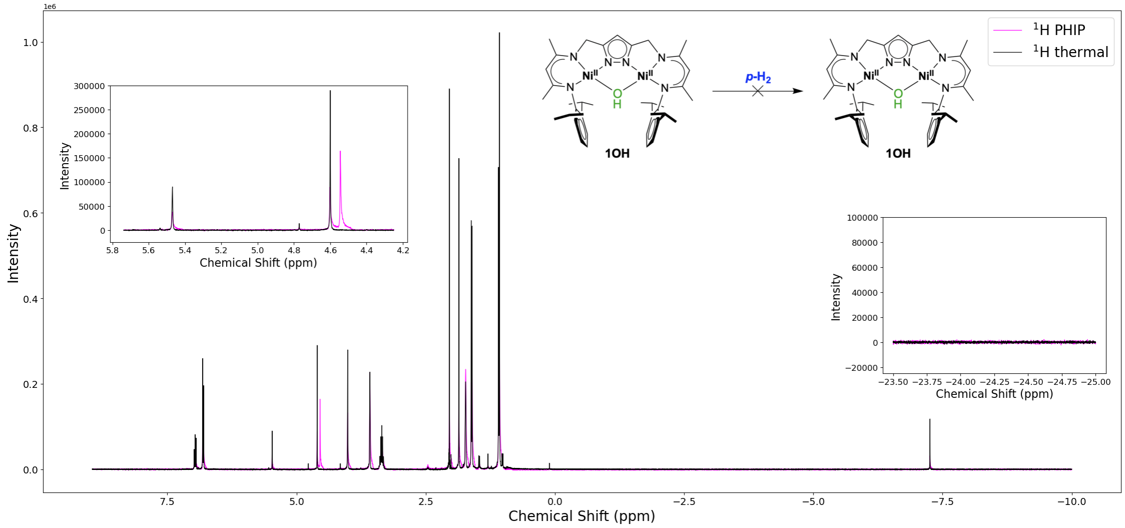

Figure S9: Overlay of 45° flip angle ^{1}H-PHIP-NMR single scan spectra of complex **1OH** (4.6 mg, 12.4 mM) in THF-d_8 (500 μL) at 298 K before (black) and after 10 s bubbling with pH$_2$ (magenta) showing no pH$_2$ induced enhancement of signals. The intensity of the single-scan spectra was scaled to the signal of THF-d_7 at 3.58 ppm. The inserts show zoom-in fractions of the spectral regions that showed sensitivity enhanced signals for bubbling experiments with complex **1H$_2$**.

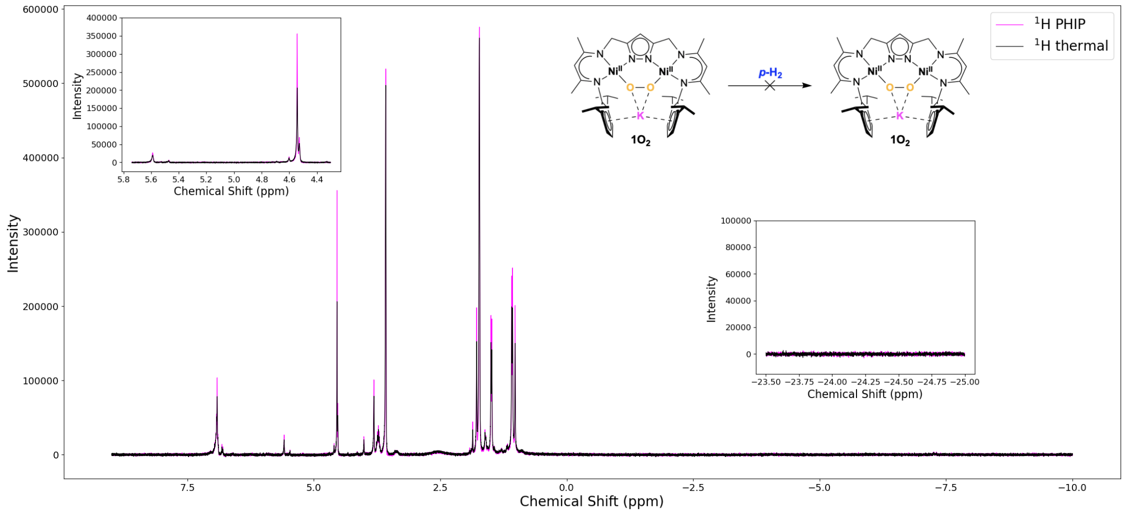

Figure S10: Overlay of 45° flip angle ^{1}H-PHIP-NMR single scan spectra of complex **1O$_2$** (approx. 2 mg, 5.05 mM) in THF-d_8 (500 μL) at 298 K before (black) and after 10 s bubbling with pH$_2$ (magenta) showing no pH$_2$ induced enhancement of signals. The inserts show zoom-in fractions of the spectral regions that showed sensitivity enhanced signals for bubbling experiments with complex **1H$_2$**.

1.5 PHIP-CEST Experiments

Samples of complex **$1H_2$** were prepared as stated above. PHIP-CEST experiments were performed with automated operation of the magnetic valves switched during the NMR pulse sequence, controlled by the TTL output signals of the spectrometer (TTL1: bypass, TTL2: pH_2, TTL3: N_2, TTL4: exhaust) according to the pulse sequence depicted in Figure S11 (pulse programm can be found in in the appendix as PHIP-CEST pulse sequence example). 24 ^{1}H NMR spectra with a 45° flip-angle were acquired. The saturation irradiation was applied for 2 s with a spin-lock amplitude of 50 Hz and central frequencies varied according to lists given below. PHIP-CEST profiles were obtained by plotting the value of the corresponding integral, either of Ni–H at −24.12 ppm or of H_2 versus the CW offset using an automated script in Python 3.0. 0th order phase correction and baseline corrections were employed using Topspin 4.0.8. and data was exported as a txt file. Further analysis of the data was performed using Python 3.0 in Jupyter Notebooks.

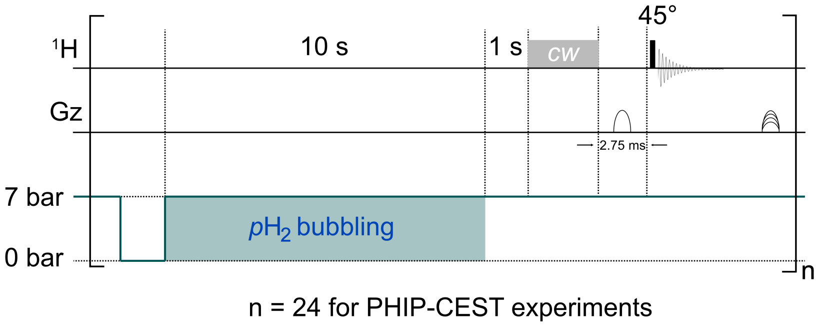

Figure S11: Pulse program used for PHIP-CEST experiments. All PHIP-CEST spectra were acquired with 45° flip-angle pulses as single-scan spectra at 9.4 T (400 MHz) using 2 s cw saturation after 10 s bubbling and allowing the sample to settle for 1 s. Spin-lock-field amplitudes ($\gamma_H B_1$) were set to 50 Hz and spin-lock-field offsets (cw offset) were varied according to linearly and non-linearly distributed lists of 24 points (n = 24) from −25 to 7.5 ppm.

Table S1: List 1 for the offset of CW saturation irradiation for PHIP-CEST experiments.

Acquisition	1	2	3	4	5	6	7	8	9	10	11	12
ν_{CW} / ppm	7.5	7.0	6.5	6.0	5.5	5.0	4.54	4.23	4	3.9	3.45	3.0
Acquisition	13	14	15	16	17	18	19	20	21	22	23	24
ν_{CW} / ppm	2.7	1	0	−1	−7	−10	−17	−23	−23.5	−24.12	−24.5	−25

Table S2: List 2 for the offset of CW saturation irradiation for PHIP-CEST experiments.

Acquisition	1	2	3	4	5	6	7	8	9	10	11	12
ν_{CW} / ppm	−25	−24.5	−24.12	−23.5	−23	−17	−16	−15	−14	−10	−7	−1
Acquisition	13	14	15	16	17	18	19	20	21	22	23	24
ν_{CW} / ppm	0	1	2.7	3	3.45	3.9	4	4.23	4.54	5.0	6.0	7.0

Table S3: List 3 for the offset of CW saturation irradiation for PHIP-CEST experiments.

Acquisition	1	2	3	4	5	6	7	8	9	10	11	12
ν_{CW} / ppm	−25	−24.12	−23	−17	−10	−7	0	1	2.7	−30	3.45	3.9
Acquisition	13	14	15	16	17	18	19	20	21	22	23	24
ν_{CW} / ppm	4	4.23	4.54	5	5.5	6	−15	6.5	−24.12	7	4.54	−25

1.5.1 Additional Discussion for PHIP-CEST Experiments

Despite the efforts made to create inert conditions for the PHIP experiments, some complex degradation can be observed during the experiments. In particular during PHIP-CEST experiments, where PHIP experiments were repeated multiple times, sample degradation lead to a loss in PHIP activity. For this reason, only a limited number of offsets were sampled, mostly centered around the hydride and the H_2 region of the spectra. In sorted lists for the offsets (lists S1 & S2), sample activity loss will result in gradual loss in sample activity along the CW offset axis, while PHIP-CEST profiles appear less scattered. In the randomized list (list S3), sample activity loss during the experiment will result in an apparent "scattering" of the data points for different offsets, which can be misleading for the interpretation of the data: The PHIP-CEST profile for the Ni−H integral shows a decrease in intensity at −15 ppm for two samples (Fig. S15 with 17.1 mM and S14 with 18.1 mM **1H$_2$ in THF-**d_8. For both PHIP-CEST experiments the CW irradiation offset was varied

according to list S3. In this list the CW irradiation at -15 ppm is at the 19th place with as many sequential 10 s parahydrogen bubbling blocks. This accounts to a total time of 190 s parahydrogen bubbling at 7 bar. At this high amount sample partial sample decomposition is very likely due to the high reactivity of $\mathbf{1H_2}$. This sample decomposition leads to a decrease of the amount of $\mathbf{1H_2}$ left in the sample. Accordingly, the expected sample activity decreases, resulting in a lower integral size for the Ni–H hydride protons. Therefore, this integral decrease is not explained by a saturation decrease due to presence of a species at -15 ppm. This is further corroborated by the experiment with the corresponding PHIP-CEST profile in Figure S13. There, the spectral region around -15 ppm was probed indirectly by irradiation at -16, -15 and -14 ppm. However, no intensity decrease is evident, suggesting that no species leading to saturation transfer is present in this spectral region.

1.6 Additional PHIP-CEST Profiles

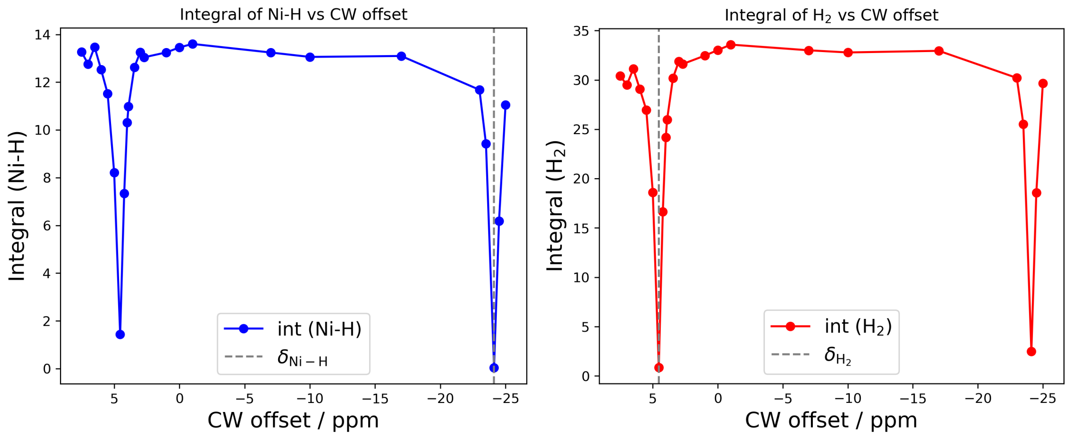

Figure S12: [1]H-PHIP-CEST profiles obtained at 9.4 T and 298 K for $\mathbf{1H_2}$ (5.1 mg, 13.4 mM) in THF-d_8 (500 μL) using 2 s CW irradiation after 10 s bubbling with pH$_2$. Spin-lock field amplitudes ($\gamma_H B_1$) were set to 50 Hz and spin-lock field offsets were varied linearly according to list S1. The value of the Ni–H integral at -24.1 ppm (blue) or H_2 integral at 4.54 ppm (red) in a.u. is plotted vs. the CW irradiation offset in ppm relative to tetramethyl silane. Sample composition before the measurement: ($\mathbf{1O_2}$/$\mathbf{1H_2}$/$\mathbf{1(H)(OH)}$/$\mathbf{1OH}$)=0/88/12/0%.

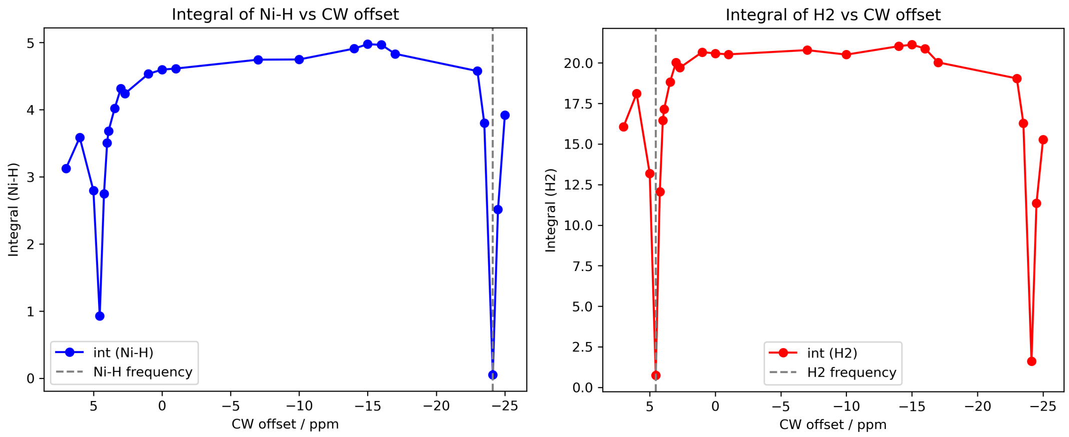

Figure S13: [1]H-PHIP-CEST profiles obtained at 9.4 T and 298 K for **$1H_2$** (5.1 mg, 13.4 mM) in THF-d_8 (500 μL) using 2 s CW irradiation after 10 s bubbling with pH$_2$. Spin-lock field amplitudes ($\gamma_H B_1$) were set to 50 Hz and spin-lock field offsets were varied linearly according to list S2. The value of the Ni−H integral at −24.1 ppm (blue) or H$_2$ integral at 4.54 ppm (red) in a.u. is plotted vs. the CW irradiation offset in ppm relative to tetramethyl silane. The sample was conducted to a second PHIP-CEST experiment before (see above), and shows lower activity due to decomposition. Sample composition before the measurement: (**$1O_2$/$1H_2$/1(H)(OH)/1OH**)=17/69/14/0%.

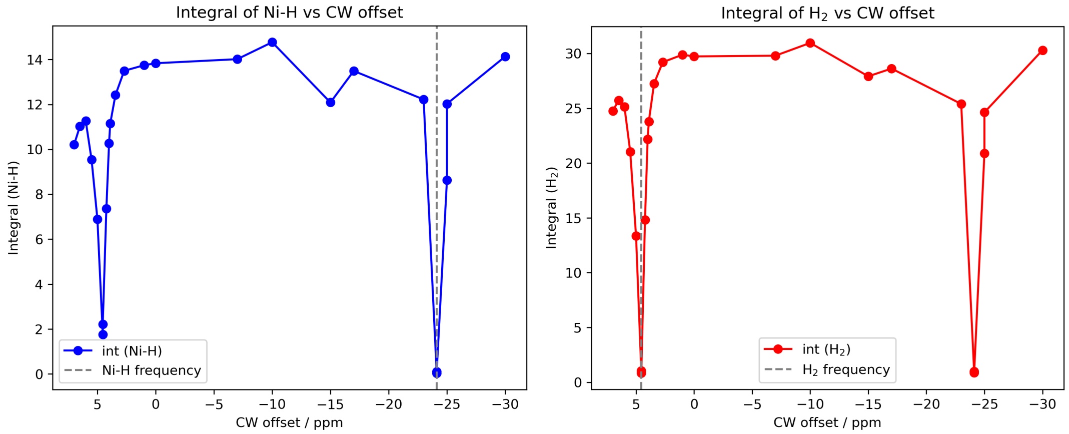

Figure S14: ^{1}H-PHIP-CEST profiles obtained at 9.4 T and 298 K for **$1H_2$** (6.9 mg, 18.1 mM) in THF-d_8 (500 μL) using 2 s CW irradiation after 10 s bubbling with pH$_2$. Spin-lock field amplitudes ($\gamma_H B_1$) were set to 50 Hz and spin-lock field offsets were varied linearly according to list S3. The value of the Ni−H integral at −24.1 ppm (blue) or H$_2$ integral at 4.54 ppm (red) in a.u. is plotted vs. the CW irradiation offset in ppm relative to tetramethyl silane. Sample composition before the measurement: (**$1O_2$/$1H_2$/1(H)(OH)/1OH**)=14/64/12/9%. The intensity dip around −15 ppm in the PHIP-CEST profile for the Ni−H integral (blue profile) is accounted to sample decomposition and not to saturation transfer. Since list S3 was used to vary the CW irradiation offset, the irradiation at −15 ppm was the 19th single scan spectrum acquired with 10 s bubbling of pH$_2$ for each spectrum, therefore sample decomposition is likely explaining the intensity decrease.

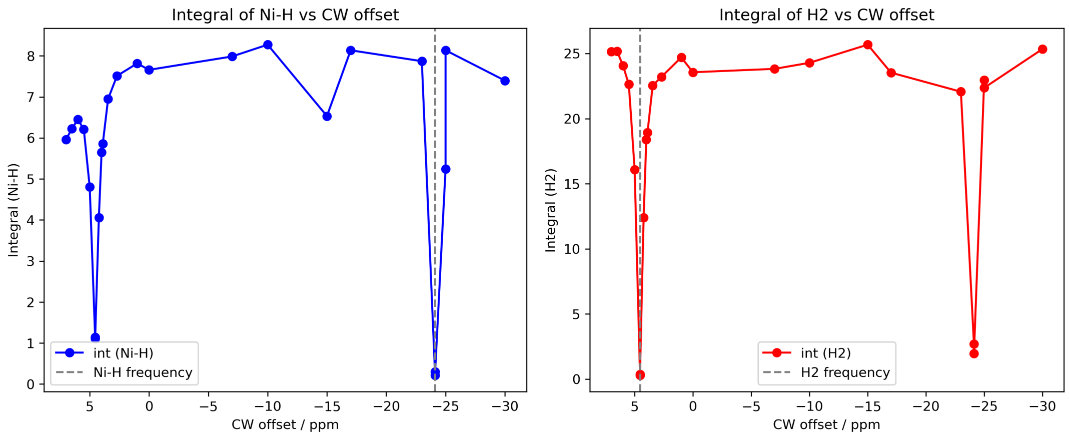

Figure S15: [1]H-PHIP-CEST profiles obtained at 9.4 T and 298 K for **$1H_2$** (6.5 mg, 17.1 mM) in THF-d_8 (500 μL) using 2 s CW irradiation after 10 s bubbling with $p$$H_2$. Spin-lock field amplitudes ($\gamma_H B_1$) were set to 50 Hz and spin-lock field offsets were varied linearly according to list S3. The value of the Ni−H integral at −24.1 ppm (blue) or H_2 integral at 4.54 ppm (red) in a.u. is plotted vs. the CW irradiation offset in ppm relative to tetramethyl silane. Sample composition before the measurement: (**$1O_2$/$1H_2$/1(H)(OH)/1OH**)=0/93/4/3%. The intensity dip around −15 ppm in the PHIP-CEST profile for the Ni−H integral (blue profile) is accounted to sample decomposition and not to saturation transfer. Since list S3 was used to vary the CW irradiation offset, the irradiation at −15 ppm was the 19th single scan spectrum acquired with 10 s bubbling of $p$$H_2$ for each spectrum, therefore sample decomposition is likely explaining the intensity decrease.

References

(S1) Manz, D.-H.; Duan, P.-C.; Dechert, S.; Demeshko, S.; Oswald, R.; John, M.; Mata, R. A.; Meyer, F. Pairwise H2/D2 Exchange and H2 Substitution at a Bimetallic Dinickel(II) Complex Featuring Two Terminal Hydrides. *J. Am. Chem. Soc.* **2017**, *139*, 16720–16731.

(S2) Duan, P.-C.; Manz, D.-H.; Dechert, S.; Demeshko, S.; Meyer, F. Reductive O2 Binding at a Dihydride Complex Leading to Redox Interconvertible -1,2-Peroxo and -1,2-Superoxo Dinickel(II) Intermediates. *J. Am. Chem. Soc.* **2018**, *140*, 4929–4939.
