# Supplementary figures and images for: Probing Hydrogen Activation in a Dimetal Dihydride Complex by Symmetric Exchange with Parahydrogen
Source: J Am Chem Soc. 2026 Feb 12;148(7):7181–8. doi: 10.1021/jacs.5c18194 (PMC12951439; doi:10.1021/jacs.5c18194)

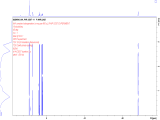

Supplement: Supplementary file 4 [file ja5c18194_si_005.zip › PHIP-CEST pulse sequence example/4/pdata/1/thumb.png]
